# Altered Intestinal Microbiomes and Lipid Metabolism in Patients With Prolonged Disorders of Consciousness

**DOI:** 10.3389/fimmu.2022.781148

**Published:** 2022-07-13

**Authors:** Jie Yu, Qisheng Cheng, Fangping He, Fanxia Meng, Yamei Yu, Chuan Xu, Xinrui Wen, Lirong Hong, Jian Gao, Jingqi Li, Gang Pan, Ming D. Li, Benyan Luo

**Affiliations:** ^1^ Department of Neurology, First Affiliated Hospital, School of Medicine, Zhejiang University, Hangzhou, China; ^2^ Department of Neurology, Sir Run Run Shaw Hospital, Zhejiang University School of Medicine, Zhejiang, China; ^3^ Department of Rehabilitation, Hangzhou Hospital of Zhejiang Armed Police Corps, Hangzhou, China; ^4^ Department of Rehabilitation, Hangzhou Mingzhou Brain Rehabilitation Hospital, Hangzhou, China; ^5^ State Key Lab of Computer Aided Design & Computer Graphics, Hangzhou, China; ^6^ State Key Laboratory for Diagnosis and Treatment of Infectious Diseases, The First Affiliated Hospital, Collaborative Innovation Center for Diagnosis and Treatment of Infectious Diseases, Zhejiang University School of Medicine, Hangzhou, China

**Keywords:** prolonged disorders of consciousness, intestinal microbiome, short-chain fatty acid, brain functional connectivity, biomarkers

## Abstract

The intestinal microbiota regulate the brain function of the host through the production of a myriad of metabolites and are associated with various neurological diseases. Understanding the intestinal microbiome of patients with prolonged disorders of consciousness (DoC) is important for the evaluation and treatment of the disease. To investigate the differences in the intestinal microbiome and short-chain fatty acids (SCFAs) among patients in a vegetative state (VS), a minimally conscious state (MCS), and emerged from MCS (EMCS), as well as the influence of antibiotics on these patients, 16S ribosomal RNA (16S rRNA) sequencing and targeted lipidomics were performed on fecal samples from patients; in addition, analysis of the electroencephalogram (EEG) signals was performed to evaluate the brain function of these patients. The results showed that the intestinal microbiome of the three groups differed greatly, and some microbial communities showed a reduced production of SCFAs in VS patients compared to the other two groups. Moreover, reduced microbial communities and five major SCFAs, along with attenuated brain functional connectivity, were observed in MCS patients who were treated with antibiotics compared to those who did not receive antibiotic treatment, but not in the other pairwise comparisons. Finally, three genus-level microbiota—*Faecailbacterium, Enterococcus*, and *Methanobrevibacter*—were considered as potential biomarkers to distinguish MCS from VS patients, with high accuracy both in the discovery and validation cohorts. Together, our findings improved the understanding of patients with prolonged DoC from the intestinal microbiome perspective and provided a new reference for the exploration of therapeutic targets.

## Background

Patients who survived severe traumatic brain injury (TBI) usually fall into prolonged disorders of consciousness (DoC), which are of two major types: vegetative state (VS) and minimally conscious state (MCS) (1–3). VS patients retain the behavioral sleep–wake cycle, but present with unawareness of oneself or the environment, whereas MCS patients show reproducible signs of awareness and exhibit fluctuations in consciousness (4, 5). In addition, patients who recover functional object use or communication from the VS or the MCS state are referred to as emerged from minimally conscious state (EMCS) (5). Different from brain-dead patients, these three types of patients retain brain activity (6) and are not dependent on a ventilator for survival. They all receive enteral nutrition. Over the last few decades, numerous evaluations and treatments have been performed for the central nervous system of patients with prolonged DoC (7). However, little attention has been paid to the peripheral physiological environment, including the intestinal microecology, which can also influence brain function.

The intestinal microbiota can modify and regulate various chemical signals from the environment through the gut–microbiota–brain axis, which then circulate throughout the body (8). Recently, it has been demonstrated that the intestinal microbiota may directly influence human health and are associated with many neurological diseases (9). In addition, short-chain fatty acids (SCFAs) are lipids produced by some intestinal microbiota through the fermentation of dietary fiber that can act on the brain by regulating neuroplasticity and the immune system (10). For example, propionic acid in the serum and feces of patients with multiple sclerosis was significantly reduced compared with that of healthy subjects; however, disability stabilization and a reduction of the annual relapse rate and brain atrophy were observed after 3 years of propionic acid intake (11). Therefore, investigation of the differences in the intestinal microbiota and the levels of SCFAs in patients with different levels of consciousness can help in understanding their association with the restoration of brain function and consciousness and discovering potential therapeutic targets related to the intestinal microbiota.

Recently, the intestinal microbiome has been widely investigated to explore the differences in the intestinal microbiota during the occurrence and development of diseases, which has substantially contributed to our understanding of patient–microbiota interactions (12). In our previous studies, high-throughput sequencing of bacterial 16S ribosomal RNA (rRNA) was used to investigate the intestinal microbiome in patients with cognitive impairment, and it was found to be closely associated with brain functional connectivity (13, 14). However, the association between patients with prolonged DoC and their intestinal microbiota still remains unexplored. To address this knowledge gap, we examined both the intestinal microbiome using 16S rRNA sequencing and SCFAs using targeted lipidomics in fecal samples specific to different levels of consciousness. Moreover, intermittent use of antibiotics is usually observed in patients with TBI for the prevention of a wide variety of infections, especially ventilator-associated pneumonia (15). Therefore, we further compared the differences in the intestinal microbiome and the brain functional connectivity between patients with prolonged DoC receiving antibiotic treatment or not, with the ultimate goal of exploring the effects of antibiotics on the intestinal microbiota of patients with different levels of consciousness and whether these alterations can influence brain function restoration and the prognosis of these patients. Finally, we identified potential biomarkers that can distinguish MCS from VS patients and EMCS from MCS patients both in the discovery and validation cohorts.

## Materials and Methods

### Ethics Statement

Written informed consent was obtained from the legal guardian of each patient. This study was approved by the Ethics Committee of the First Affiliated Hospital, School of Medicine (no. NCT04530968), Zhejiang University, Hangzhou Hospital of Zhejiang Armed Police Corps, China, and Hangzhou Mingzhou Brain Rehabilitation Hospital.

### Subjects

The participants of this study are patients with severe TBI recruited from the rehabilitation units of Hangzhou Hospital of Zhejiang Armed Police Corps, China, and Hangzhou Mingzhou Brain Rehabilitation Hospital. These patients were randomly assigned into the discovery cohort (VS: *n* = 13, average age = 50.7 ± 13.2 years, 9 men and 4 women; MCS: *n* = 15, average age = 49.0 ± 9.8 years, 10 men and 5 women; and EMCS: *n* = 15, average age = 47.7 ± 12.1 years, 12 men and 3 women) and the validation cohort (VS: *n* = 14, average age = 57.5 ± 10.5 years, 10 men and 4 women; MCS: *n* = 13, average age = 56.3 ± 14.2 years, 10 men and 3 women; and EMCS: *n* = 17, average age, 53.0 ± 16.7 years; 11 men and 6 women). The Glasgow Coma Scale (GCS) was used to assess the severity of brain injury in the acute stage, and the Glasgow Outcome Scale—Expended (GOS-E) was used to assess the prognosis of patients at 6 months (16). Diagnosis of VS, MCS, or EMCS was based on the five assessments within 10 days by DoC experts using the Coma Recovery Scale—Revised (CRS-R) (5). In brief, VS patients can open their eyes and preserve sleep–wake cycles, but are unaware of themselves and their surroundings, MCS patients show reproducible signs of awareness and exhibit fluctuations in consciousness, while EMCS patients exhibit recovery of functional object uses or communication from prolonged DoC. All patients maintained this physiological state more than 1 month prior to enrollment and accepted the diet provided in the hospital for a long period; all patients received enteral nutrition from the hospital.

Patients with other causes of DoC and unstable consciousness state (there are signs of spontaneous recovery or deterioration within 1 week) were excluded from this study. We also excluded patients who had frequent and irregular seizures, as well as those who had been taking sedatives for a long time and continuously. We also excluded the following subjects: 1) had a history of using antibiotics, probiotics, prebiotics, or synbiotics within 1 month before fecal sample collection; 2) those with severe malnutrition, infection, and drug or alcohol addiction, irritable bowel syndrome, and inflammatory bowel disease in the last year; and 3) those with schizophrenia, schizoaffective disorder, or primary affective disorder and combined severe heart, brain, liver, kidney, and hematopoietic system diseases or other serious primary diseases. Patients who received special treatments, such as transcranial magnetic stimulation (TMS) and transcranial direct current stimulation (tDCS), were also excluded. The demographic and clinical characteristics of the study participants are listed in **Table 1** and **Supplementary Table S5**.

**Table 1:** Clinical characteristics of the patients in this study.

	Without antibiotic treatment				With antibiotic treatment			
	EMCS	MCS	VS	*p*-value	EMCS-Abx	MCS-Abx	VS-Abx	*p*-value
Patients (*n*)	15	15	13	–	9	17	21	–
Men, *n* (%)	12 (80.0)	10 (66.7)	9 (69.2)	0.691	5 (55.6)	12 (70.6)	15 (71.4)	0.668
Age (years)	52.7 ± 10.2	49.0 ± 9.8	58.3 ± 9.7	0.058	61.0 ± 11.6	53.4 ± 6.5	54.9 ± 11.8	0.058
GCS	7.1 ± 1.4	6.7 ± 1.2	7.0 ± 1.6	0.707	6.1 ± 1.2	6.9 ± 1.0	7.1 ± 1.5	0.114
CRS-R	22.4 ± 0.8	11.7 ± 3.1	6.0 ± 1.3	<0.001*	22.1 ± 1.4	11.9 ± 2.9	5.2 ± 1.3	<0.001*
GOS-E	3.3 ± 0.5	2.7 ± 0.8	2.1 ± 0.6	<0.001*	3.0 ± 0.0	2.1 ± 0.6	2.1 ± 0.4	<0.001*
Length of hospital stay (days)	172.5 ± 124.9	158.7 ± 92.5	101.0 ± 55.5	0.155	79.7 ± 49.6	120.3 ± 112.3	169.5 ± 142.7	0.248
Leukocytes (10^9^/L)	5.4 ± 1.7	6.1 ± 2.0	6.5 ± 1.8	0.184	6.0 ± 1.4	6.5 ± 2.1	6.3 ± 2.1	0.817
Neutrophils (10^9^/L)	3.7 ± 1.5	4.2 ± 1.8	4.3 ± 1.4	0.503	3.7 ± 1.2	4.7 ± 2.0	4.4 ± 1.8	0.415
Lymphocytes (10^9^/L)	1.3 ± 0.4	1.3 ± 0.4	1.3 ± 0.4	0.931	1.4 ± 0.5	1.3 ± 0.5	1.2 ± 0.5	0.649
Monocytes (10^9^/L)	0.4 ± 0.2	0.4 ± 0.2	0.5 ± 0.2	0.134	0.5 ± 0.1	0.5 ± 0.3	0.7 ± 1.2	0.805
Hemoglobin (g/L)	124.3 ± 14.9	124.9 ± 16.4	111.8 ± 10.3	0.045*	117.0 ± 10.9	105.5 ± 12.5	115.5 ± 13.3	0.030*
Platelets (10^9^/L)	219.8 ± 45.9	199.1 ± 71.3	236.4 ± 58.3	0.263	209.2 ± 80.9	252.6 ± 89.0	221.5 ± 70.0	0.335
C-reactive protein (mg/L)	3.5 ± 3.7	3.2 ± 3.3	5.5 ± 4.3	0.142	4.0 ± 4.4	6.7 ± 4.0	4.6 ± 3.5	0.133
GPT (IU/L)	40.7 ± 31.5	39.3 ± 14.6	37.6 ± 25.4	0.601	24.3 ± 10.9	24.2 ± 14.2	27.3 ± 15.6	0.775
GOT (IU/L)	21.1 ± 10.6	21.3 ± 6.0	22.1 ± 10.0	0.677	18.9 ± 6.6	22.1 ± 14.9	21.3 ± 15.3	0.880
LDH (IU/L)	172.1 ± 34.5	201.4 ± 62.7	165.4 ± 49.0	0.135	159.3 ± 38.3	168.4 ± 59.8	153.5 ± 42.9	0.831
Creatinine (μmol/L)	50.7 ± 16.1	47.2 ± 12.9	36.7 ± 10.0	0.025*	43.4 ± 6.9	44.5 ± 19.1	38.2 ± 11.1	0.279
Homocysteine (μmol/L)	12.0 ± 2.2	9.9 ± 3.5	10.9 ± 1.6	0.101	9.6 ± 2.2	7.8 ± 2.9	8.7 ± 3.9	0.370
Cause	TBI	TBI	TBI	/	TBI	TBI	TBI	/

Continuous variables are expressed as the mean ± SD. A * was considered statistically significant.

GCS, Glasgow Coma Scale; CRS-R, Coma Recovery Scale—Revised scores; GOS-E, Glasgow Outcome Scale—Extended; GPT, glutamic–pyruvic transaminase; GOT, glutamic–oxaloacetic transaminase; LDH, lactate dehydrogenase; TBI, traumatic brain injury; VS, vegetative state; MCS, minimally conscious state; EMCS, emerged minimal conscious state; Abx, antibiotic.

Three other groups of patients who had a history of antibiotic use but conform to the other exclusion criteria were also recruited from these same hospitals: VS with antibiotics (VS-Abx) (*n* = 21, average age = 54.9 ± 11.8 years, 15 men and 6 women); MCS with antibiotics (MCS-Abx) (*n* = 17, average age = 62.8 ± 8.7 years, 12 men and 5 women); and EMCS with antibiotics (EMCS-Abx) (*n* = 9, average age = 61.0 ± 11.6 years, 5 men and 4 women). Some of the patients with DoC briefly received prophylactic antibiotic treatment to prevent aspiration pneumonia. Others continued treatment for a short period to prevent reinfection, even if the previous infection had been suppressed. The demographic and clinical characteristics of the patients are listed in **Table 1**.

All study participants were categorized based on their CRS-R scores, and centrally acting drugs, neuromuscular function blockers, and sedation were discontinued for at least 24 h when the samples were collected (17). Fasting plasma samples from all patients were collected on the day of fecal sample collection. Patients’ intact skulls were also accepted for resting EEG recording.

### Sample Collection and Extraction of Genome DNA

Fecal samples from EMCS, MCS, and VS patients were collected at admission and were processed in the laboratory within 4 h after collection. All fecal samples were dispensed in 2-ml Eppendorf tubes within half an hour, each tube packing 180 ± 20mg, and immediately stored at −80°C until analysis. Total genome DNA was extracted from the samples using the cetyltrimethylammonium bromide (CTAB) and sodium dodecyl sulfate (SDS) method (18). The DNA concentration and purity were monitored on 1% agarose gels. Based on the concentration, DNA was diluted to 1 ng/µl using sterile water.

### Amplicon Generation, Polymerase Chain Reaction, and Sequencing

The isolated bacterial genomic DNA was used as a template for the PCR amplification of the V3–V4 regions of the bacterial 16S rRNA gene in a multiplex approach with forward primers. All PCR reactions were carried out with 15 µl of Phusion^®^ High-Fidelity PCR Master Mix (New England Biolabs, Ipswich, MA, USA), 0.2 µM of forward and reverse primers, and about 10 ng of template DNA. Thermal cycling consisted of an initial denaturation at 98°C for 1 min, followed by 30 cycles of denaturation at 98°C for 10 s, annealing at 50°C for 30 s, and extension at 72°C for 30 s, and finally at 72°C for 5 min. The same volume of 1× loading buffer (containing SYB green) was mixed with the PCR products and electrophoresis performed on 2% agarose gel for detection. The PCR products were mixed in equidensity. Then, the mixture was purified with a Qiagen Gel Extraction Kit (Qiagen, Hilden, Germany). The sequencing libraries were generated using the TruSeq^®^ DNA PCR-Free Sample Preparation Kit (Illumina, San Diego, CA, USA) following the manufacturer’s recommendations, and index codes were added. The library quality was assessed on the Qubit@ 2.0 Fluorometer (Thermo Scientific, Waltham, MA, USA) and the Agilent Bioanalyzer 2100 system. Lastly, the library was sequenced on an Illumina NovaSeq platform and 250-bp paired-end reads were generated.

### Short-Chain Fatty Acid Measurements

The samples were thawed on ice and diluted at 1:10 in sterile phosphate-buffered saline (PBS) solution, vortexed for 1 min, and centrifuged at 1,000 × *g* for 2 min at 4°C. As described in our previous study (19, 20), the supernatant was recovered and the pellet extracted with isopropyl ether. Aliquots (100 µl) were added into a 2-ml glass centrifuge tube and mixed with 50 μl of water with 15% phosphoric acid and 150 µl of 5 µg/ml 4-methyl valeric acid (internal standard, IS). The suspensions were homogenized with a vortex for about 1 min and centrifuged for 10 min at 12,000 × *g*. Of the supernatant, 1 μl was taken for GC-MS analysis using an Agilent Model 7890A/5975C GC-MS system. To quantify the SCFAs, a calibration curve for the concentration range 0.1–100 µg/ml was constructed. The IS was used to correct for injection variability between samples and for minor changes in the instrument response.

The samples were separated with an Agilent HP-INNOWAX capillary GC column (30 m × 0.25 mm ID × 0.25 µm). The initial temperature was 90°C, which was increased to 120°C at 10°C/min, after which it was increased to 150°C at 5°C/min and then to 250°C at 25°C/min, where it remained for 2 min. The carrier gas was helium (1.0 ml/min). The temperatures of the injection port and the transmission line were 250°C and 230°C, respectively. The electron bombardment ionization source, selected ion monitor (SIM) scanning mode, and the electron energy was 70 eV.

### Data Processing

Sequence analysis was performed using Uparse software (21). All reads were deposited and grouped into operational taxonomic units (OTUs) at a sequence identity of 97%, and the taxonomic affiliation of the OTUs was determined with quantitative insights into microbial ecology (QIIME, version 1.8.0) against the Greengenes database, version 13.8 (22). The following downstream data analyses were conducted in R software. Alpha diversity was applied in analyzing the complexity of species diversity for a sample through four indices, namely, Chao1, Shannon, Simpson, and ACE (abundance-based coverage estimator). All these indices were calculated for the samples with QIIME (version 1.7.0) and displayed using R software (version 2.15.3). Principal coordinate analysis (PCoA) used unweighted and weighted UniFrac distance matrices and the Bray–Curtis distance matrix (23). The linear discriminant analysis (LDA) effect size (LEfSe) method was used to characterize the taxa with statistical significance and biological relevance. For LEfSe analysis, the Kruskal–Wallis test (alpha value of 0.05) and an LDA score >4 were used as thresholds. Pairwise comparisons were analyzed with the Mann–Whitney *U* test. Partial least squares latent structure discriminant analysis (PLS-DA) was performed using MetaboAnalyst 4.0 (www.metaboanalyst.ca) to observe the fecal microbiota structure in the different groups (13) based on the OTUs of the sequencing data from each sample. Variable importance in projection (VIP) reflects the importance of the variables that have the most significant contribution in the discrimination. Variables with VIP >1 are important contributors to the generation of the model. Based on Kyoto Encyclopedia of Genes and Genomes (KEGG) functional pathways, Phylogenetic Investigation of Communities by Reconstruction of Unobserved States (PICRUSt) (24) was used to predict the functional composition of the intestinal microbiome for each sample.

### EEG Data Recording and Analysis

EEG signals were recorded using a 64-electrode BrainCap (Brain Products GmbH, Munich, Germany) in the International 10–20 system, and 1 of the 64 electrodes was placed under the right eye to record the electrooculogram (EOG). The electrode impedances were maintained below 10 kΩ. The signals were sampled at 1 kHz. EEG signals were referenced online to FCz (mid-frontal), but were referenced offline to a common average reference (25). Raw EEG data were digitally filtered between 0.1 and 46 Hz (bandpass filter). Baseline correction was also applied to all channels. EEG epochs with ocular, muscular, and other artifacts were visually identified and manually rejected (26). This was followed by an independent component analysis (ICA; InfoMax algorithm) to remove periodically recurring artifacts, including horizontal eye movements, blinks, and ECG artifacts. All preprocessing and analyses in this study were conducted in MATLAB software (The MathWorks, Natick, MA, USA).

A frequency spectrum was computed. An EEG power spectrum density (PSD) was generated using the Welch’s method (one of the Fourier transforms) (26). The different frequency bands on the frequency spectrum were divided by 0.1–46 Hz to obtain the relative power values. The power values were then averaged across each frequency band—delta (1–4 Hz), theta (4–8 Hz), alpha (8–12 Hz), beta (12–30 Hz), and gamma (31–45 Hz)—and were averaged across all trials.

Functional connectivity assesses the functional communication between the brain regions by estimating the level of synchronization of the EEG signals (27). The phase lag index (PLI) aims to obtain a measure that provides reliable estimates of the phase synchronization between two signals and is insensitive to volume conduction (28). Here, the instantaneous phases were obtained by initially bandpass filtering the signals within the frequency bands defined above and subsequently using the Hilbert transform to obtain the phase of the corresponding analytic signal (28, 29). The phase difference distribution (1*φ*), as an index of asymmetry between a given pair of channels that were wrapped in the interval, can be obtained as follows:


$${\text{PLI}}_{xy}=\left|\langle \text{sgn}\left(\Delta \varphi \left(\tau \right)\right)\rangle \right|$$

Different from PLI, the weighted phase lag index (WPLI) is an improved measure of phase synchronization for electrophysiological signals in the presence of noise and volume conduction, and it calculates the weight of the contribution of the observed phase leads and lags by the magnitude of the imaginary component of the cross-spectrum (28).


$$\text{WPLI }=\frac{\left|\langle \text{Imag}\left(Sxy\left(f\right)\right)\rangle \right|}{\langle \left|\text{Imag}\left(Sxy\left(f\right)\right)\right|\rangle }=\frac{|<\left|\text{Imag}\left(Sxy\left(f\right)\right)\right|\cdot \text{sgn}\left(\text{Imag}\left(f\right)\right)>|}{\langle \left|\text{Imag}\left(Sxy\left(f\right)\right)\right|\rangle }$$

The PLI and WPLI range between 0 and 1, with 0 indicating no correlation and 1 maximal correlation. The connectivity matrices were entered as repeated measures dependent variables into the network-based statistic (NBS) toolbox (30). All of the analyses were conducted in MATLAB software (The MathWorks, Natick, MA, USA).

### Statistical Analysis

Continuous variables were reported as the mean ± SEM, and statistical comparisons were made using one-way analysis of variance (ANOVA) followed by *post-hoc* least significant difference (LSD) or an independent *t*-test. Non-normally distributed variables were expressed as interquartile ranges (IQRs), and comparisons were conducted using the Mann–Whitney *U* test, Kruskal–Wallis test, or the chi-square test. Receiver operating characteristic (ROC) analyses and least absolute shrinkage and selection operator (LASSO) regression were constructed to calculate the best cutoff points and areas under the curve (AUCs) for the candidate biomarkers. For correlation analysis, Spearman’s rank test was performed. Statistical analysis was performed using SPSS version 23.0. *P*-values less than 0.05 after multiple comparison correction using the false discovery rate method were considered significant.

## Results

### Clinical Characteristics of the Study Cohorts

In this study, 43 participants without antibiotic intervention and 47 participants with antibiotic intervention, including EMCS, MCS, and VS patients, were recruited for 16S rRNA sequencing of fecal samples. The demographic and clinical characteristics of the study cohorts are summarized in **Table 1**. As shown, there were no significant differences among the three groups with respect to age, sex, and length of hospital stay. We also recorded and compared the main biochemical indicators of patients on the day of fecal sample collection. No significant differences were found in these indicators, excluding the levels of hemoglobin and creatinine among the three groups with or without antibiotic intervention.

In order to exclude the influence of the severity of brain injury in the acute stage, we compared the GCS of the patients when they were admitted in the ICU and found no significant differences among the three groups with or without antibiotic use.

### Alterations in the Microbiomes of VS, MCS, and EMCS Patients Without Antibiotic Treatment

**Figure 1:**
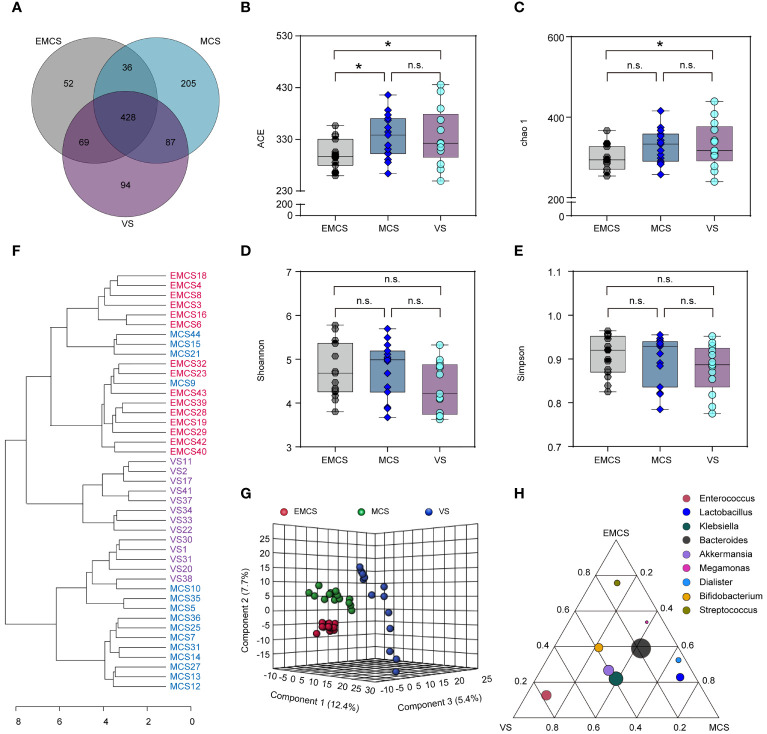
Changes in the fecal microbial diversity and community structures of EMCS, MCS, and VS patients. **(A)** Venn diagram showing the overlap of the operational taxonomic units of the fecal microbiota across the three groups. **(B–E)** The α-diversity of the fecal microbiomes of three groups depicted according to ACE, Chao1, Shannon, and Simpson indices. Each box plot represents the median, interquartile range, minimum, and maximum values. **(F)** Dendrogram of the EMCS, MCS, and VS groups showing the clustering of all 43 samples. **(G)** PLS-DA score plots of the relative abundances of the microbiota in the EMCS, MCS, and VS groups. Three components explained 25.5% (12.4%, 5.4%, and 7.7%) of the variance among the three groups. Component scores are indicated as percent. Circles indicate individual samples from the EMCS, MCS, and VS groups.**(H)** Ternary plot depicting the number of genera (top 10 relative abundance) enriched in the EMCS, MCS, and VS groups. Each *circle* represents one genus, and the *size of the circle* reflects the relative abundance. *P*-values were determined using one-way ANOVA or the Kruskal–Wallis test. *p < 0.05; *n.s.*, no significant difference. *EMCS*, emerged from minimally conscious state; *MCS*, minimally conscious state; *VS*, vegetative state; *PLS-DA*, partial least squares discriminant analysis.

To assess alterations in the intestinal microbiome of EMCS, MCS, and VS patients, 16S rDNA gene sequencing was performed on all samples. As shown in the Venn diagram, 971 OTUs were identified, of which 428 OTUs were shared among all samples. MCS and VS patients shared 515 of 919 OTUs, EMCS and VS patients shared 497 of 766 OTUs, and EMCS and MCS patients shared 464 of 877 OTUs (**Figure 1A**). The mean community richness values (ACE and Chao1 indices) were higher in the VS and MCS groups compared to the EMCS group (**Figures 1B, C**). However, there was no significant difference in the microbial diversity (Shannon and Simpson diversity indices) among the three groups (**Figures 1D, E**). Subsequently, we applied feature selection to identify the gut microbiota that maximized the identification of the EMCS, MCS, and VS groups *via* PLS-DA (*R* ^2^ = 0.998, *Q* ^2^ = 0.842) (**Figure 1G**). We also performed PCoA and cluster analysis (**Supplementary Figure S1A** and **Figure 1F**), the results of which were consistent with those of PLS-DA: the samples from the MCS group were located between the EMCS and VS groups, but were closer to those of the EMCS group. Furthermore, we identified the importance of fecal microbiota in the PLS-DA model based on the VIP score, which is the weighted sum of squares of the PLS loadings for that variable (19). Thirty-eight OTUs were identified based on a VIP score >2 (**Supplementary Figure S1B**). Of the fecal microbiota, *Lactococcus*, *Megamonas*, and *Enterococcus*, among others, were enriched in the VS group, while *Allisonella*, *Mogibacterium*, and *Campylobacter*, among others, were enriched in the EMCS group; however, most of the intestinal microbiota showed lower levels in the MCS group compared to the other two groups (**Supplementary Figure S1C**). Furthermore, we screened the top 10 OTUs with the highest relative abundance using a ternary plot. *Streptococcus* was enriched in EMCS patients, *Lactobacillus* was enriched in MCS patients, and *Megamonas* was enriched in VS patients (**Figure 1H**), suggesting that the interior structure of the intestinal microbiome was also altered in patients with different levels of consciousness.

### Fecal Microbiota Taxonomic Differences Among EMCS, MCS, and VS Patients Without Antibiotic Treatment

**Figure 2:**
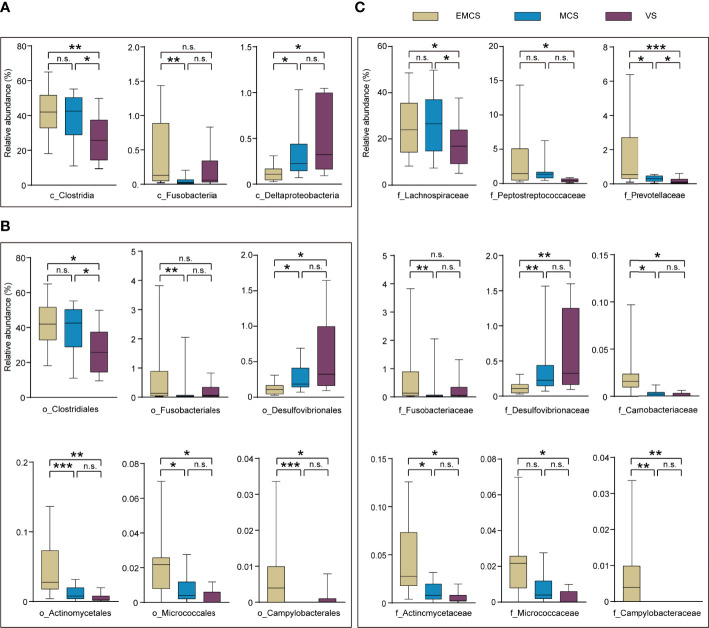
Taxonomic differences of the class, family, and order levels in the fecal microbiota among EMCS, MCS, and VS patients. **(A–C)** Comparison of the relative abundance rates at the class level **(A)**, order level **(B)**, and family level **(C)** across the three groups. Each *box* represents an interquartile range (first and third quartiles) of taxon abundance, and the *middle line* corresponds to the median abundance. *No box* indicates that the fecal microbiota cannot be detected in the group. *P*-values were corrected using FDR. *p < 0.05, **p < 0.01, ***p < 0.001; *n.s.*, no significant difference. *c*, class; *f*, family; *o*, order; *EMCS*, emerged from minimally conscious state; *MCS*, minimally conscious state; *VS*, vegetative state; *FDR*, false discovery rate.

**Figure 3:**
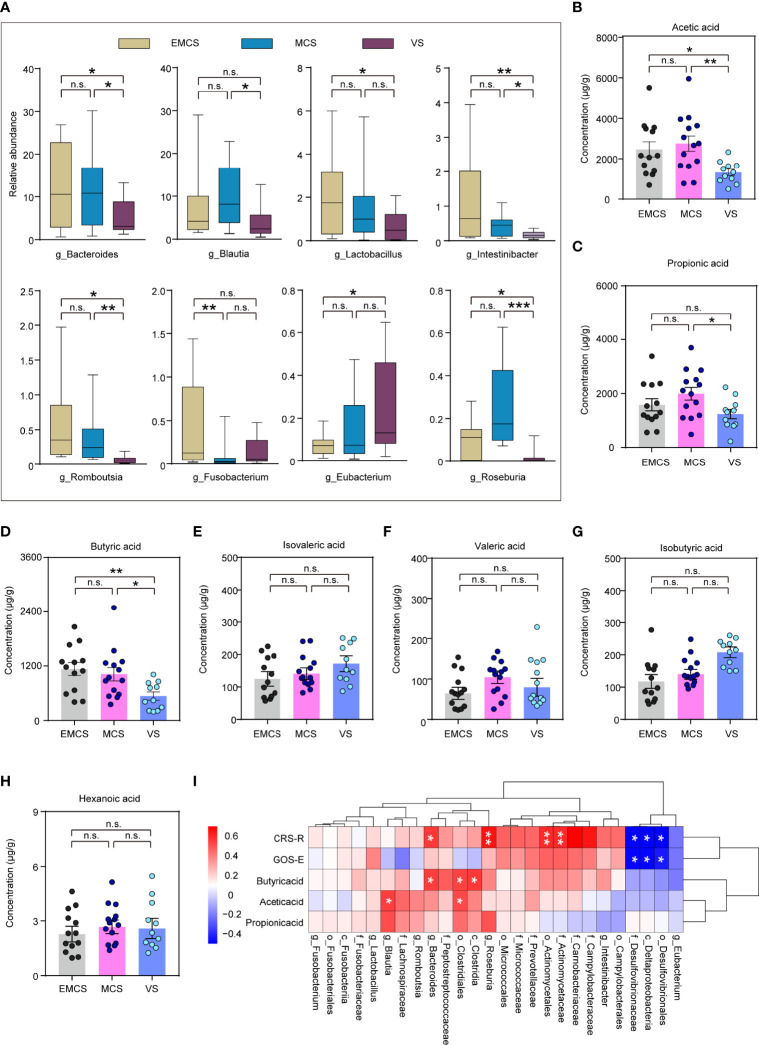
Taxonomic differences at the genus level and differences in the short-chain fatty acids (SCFAs) in the fecal microbiota of EMCS, MCS, and VS patients. **(A)** Comparison of the relative abundance at the genus level across the three groups. Each *box* represents an interquartile range (first and third quartiles) of taxon abundance, and the *middle line* corresponds to the median abundance. *P*-values were corrected using FDR. **(B–H)** Comparison of the concentrations of the differential fecal SCFAs across the three groups. Data represent the mean ± SEM. **(I)** Heatmap of Spearman’s rank correlation analysis between the altered fecal microbiota and SCFAs and the CRS-R scores. *Red* indicates positive correlation and *blue* denotes negative correlation. *P*-values were determined using one-way ANOVA or the Kruskal–Wallis test. *p < 0.05, **p < 0.01, ***p < 0.001; *n.s.*, no significant difference. *g*, genera; *CRS-R*, Coma Recovery Scale—Revised scores; *GOS-E*, Glasgow Outcome Scale—Extended; *EMCS*, emerged from minimally conscious state; *MCS*, minimally conscious state; *VS*, vegetative state; *FDR*, false discovery rate.

To investigate specific alterations in the fecal microbiota of patients with different levels of consciousness, we assessed the relative abundances in the EMCS, MCS, and VS groups at the phylum, class, family, and genus levels (**Figures 2**, **3A**). In our study, none of the fecal microbiota at the phylum level showed significant differences among the three groups. At the class level, VS patients showed a marked decrease in the relative abundance of Clostridia compared to the MCS and EMCS groups, while the relative abundance of Fusobacteriia showed alterations only between the MCS group and the EMCS group. Conversely, Deltaproteobacteria, the intestinal microbiota associated with inflammatory response (31), showed a higher relative abundance in the VS group than in the other two groups (**Figure 2A**), which indicated that the intestinal microenvironment of VS patients may be stimulated by chronic inflammation for a long period. Specifically, the subcategories Clostridiales and Lachnospiraceae and three genera—*Intestinibacter*, *Romboutsia*, and *Roseburia—*showed similar significant alterations in the microbiome of VS patients, which mostly explained the decreased abundance of Clostridia. Unexpectedly, *Eubacterium*, which is related to the production of intestinal butyric acid (32), showed a higher relative abundance in VS patients compared to EMCS patients. Similar significance was also found for Fusobacteriia, Deltaproteobacteria, and their corresponding order and family levels. In addition, Actinomycetaceae, Micrococcaceae, and Campylobacteraceae, which explained their order levels, showed higher relative abundance rates in the EMCS group than in the other two groups (**Figures 2B, C**). At the genus level, most of the intestinal microbiota that produced SCFAs or branched-chain fatty acids, such as *Bacteroides*, *Blautia*, *Lactobacillus*, *Roseburia*, and *Fusobacterium* (10, 33, 34), showed dramatically low abundance rates in patients in MCS or VS (**Figure 3A**).

### Fecal SCFA Differences Among EMCS, MCS, and VS Patients Without Antibiotic Treatment

SCFAs regulate the activity of neural cells and confer benefits for many neurological diseases through the gut–microbiota–brain axis (11, 35). To further explore the SCFAs in the intestinal tract of the patients in the three groups, they were also determined in the same fecal samples using targeted lipidomics. The results showed that the concentrations of acetic acid, propionic acid, and butyric acid were significantly decreased in the VS group (**Figures 3B–D**), which were consistent with the differences in plasma reported in our previous study (19). Other fatty acids such as isobutyric, isovaleric, valeric, and hexanoic acids were not significantly different among the three groups (**Figures 3E–H**). In the correlation analysis, *Bacteroides*, Clostridiales, and *Blautia* were significantly positively correlated with acetic acid or propionic acid. Moreover, some fecal microbiota including valeric acid-associated bacteria (Actinomycetaceae), *Roseburia*, and *Bacteroides* were significantly positively correlated with the CRS-R scores of patients, whereas the pathogenic microbial community (Desulfovibrionaceae) showed significant negative correlations with both the CRS-R and GOS-E scores (**Figure 3I**). This suggested the existence of a causal relationship between the disturbance of the microbial community and posttraumatic prolonged DoC, which may result from the reduction of the levels of SCFAs and the elevation of the pathogenicity.

### Predicted Function Analysis of the Microbiome in EMCS, MCS, and VS Patients Without Antibiotic Treatment

To further predict the functional composition of the intestinal microbiome of prolonged DoC patients, PICRUSt2 was employed. **Table 2** shows the top 10 differential pathways between MCS and VS patients. Broad potential communication lines were identified between the gut microbiome and metabolism in EMCS, MCS, and VS patients, including folate transformation and l-threonine, l-arginine, l-tryptophan, and l-aspartate biosynthesis. Consistent with the trends in the PLS-DA and PCoA models, all of these pathways were significantly higher in the EMCS and MCS groups compared to the VS group. Some of these pathways, such as L-threonine biosynthesis, L-aspartate biosynthesis, and folate transformation, were also significantly higher in the EMCS group compared to the MCS group, suggesting that the synthesis of some essential amino acids in patients with low levels of consciousness may be weak, which is consistent with our previous metabolomics findings (19).

**Table 2:** Pathway analysis of the EMCS, MCS, and VS groups using PICRUSt2.

Pathway	EMCS mean% (SD%)	MCS mean% (SD%)	VS mean% (SD%)	*p-*values					
				EMCS *vs*. MCS	EMCS *vs*. VS			MCS *vs*. VS
Gondoate biosynthesis	0.827 ± 0.088	0.836 ± 0.080	0.741 ± 0.059	0.782			0.005*	0.001*
*cis*-Vaccenate biosynthesis	0.768 ± 0.078	0.769 ± 0.068	0.701 ± 0.053	0.994			0.013*	0.007*
l-Threonine biosynthesis	0.681 ± 0.054	0.680 ± 0.061	0.609 ± 0.056	0.973			0.002*	0.004*
Folate transformations III	0.627 ± 0.046	0.625 ± 0.048	0.570 ± 0.047	0.901			0.003*	0.005*
l-Arginine biosynthesis II	0.537 ± 0.071	0.585 ± 0.061	0.510 ± 0.069	0.061			0.317	0.006*
l-Arginine biosynthesis I	0.540 ± 0.066	0.580 ± 0.053	0.503 ± 0.065	0.076			0.152	0.002*
l-Arginine biosynthesis IV	0.543 ± 0.000	0.579 ± 0.052	0.502 ± 0.064	0.079			0.138	0.002*
l-Tryptophan biosynthesis	0.528 ± 0.098	0.555 ± 0.067	0.481 ± 0.070	0.380			0.150	0.008*
l-Aspartate and l-asparagine biosynthesis	0.529 ± 0.071	0.520 ± 0.061	0.450 ± 0.066	0.724			0.005*	0.007*
Flavin biosynthesis	0.483 ± 0.091	0.529 ± 0.066	0.433 ± 0.051	0.128			0.080	<0.001*

Data are given as mean% (SD%). A * was considered statistically significant.

VS, vegetative state; MCS, minimally conscious state; EMCS, emerged minimal conscious state; PICRUSt, Phylogenetic Investigation of Communities by Reconstruction of Unobserved States.

### Fecal Microbiota Taxonomic and SCFA Differences Between Prolonged DoC Patients With and Without Antibiotic Treatment

**Figure 4:**
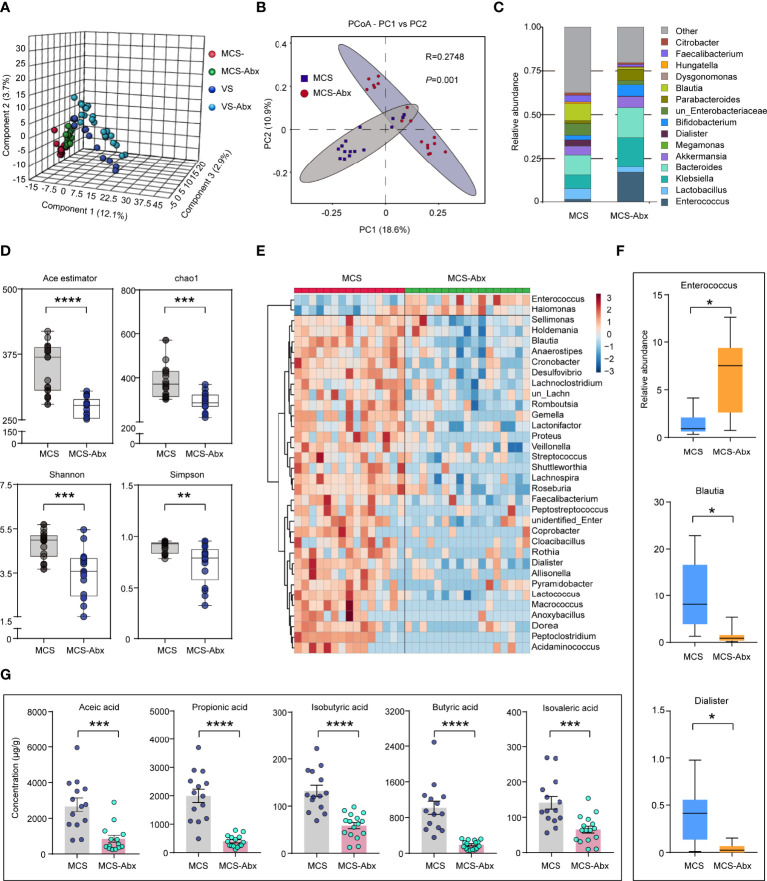
Comparison of the fecal microbial diversity and community structures in MCS patients with or without antibiotic treatment. **(A)** PLS-DA score plots of the relative abundances of the microbiota in the MCS and MCS-Abx groups and in the VS and VS-Abx groups. Three components explained 18.7% (12.1%, 2.9%, and 3.7%) of the variance among the four groups. Component scores are indicated as percent. *Circles* indicate individual samples from the four groups. **(B)** Beta diversity results of the MCS and MCS-Abx groups assessed using principal coordinate analysis (PCoA). A total of 32 samples were used for PCoA. Two principal components (PCs) explained 29.5% (18.6% and 10.9%) of the variance between the groups (Bray–Curtis distance: MCS *vs*. MCS-Abx: *R* = 0.2748, *p* = 0.001). PC scores are indicated as percent. *Circles* indicate individual samples from the MCS and MCS-Abx groups. **(C)** Taxonomic distributions of the fecal microbiota at the genus level in the MCS and MCS-Abx groups. **(D)** The α-diversity of the fecal microbiomes between the MCS and MCS-Abx groups depicted according to ACE, Chao1, Shannon, and Simpson indices. Each *box plot* represents the median, interquartile range, minimum, and maximum values. **(E)** Heatmap of the most abundant metabolites in the MCS and MCS-Abx groups, as identified by the VIP scores in the PLS-DA. Each sample represents a single column. *Red* indicates the greater abundance of metabolites. **(F)** Top 3 differential fecal microbiota at the genus level by MetaStat analysis. Each *box* represents an interquartile range (first and third quartiles) of taxon abundance, and the *middle line* corresponds to the median abundance. *P*-values were corrected using FDR. **(G)** Comparison of the concentrations of the differential fecal short-chain fatty acids between the MCS and MCS-Abx groups. Data represent the mean ± SEM. *P*-values were determined using Student’s *t*-test or the Kruskal–Wallis test and were corrected using FDR. *p < 0.05, **p < 0.01, ***p < 0.001, ****p < 0.0001; *n.s.*, no significant difference. *MCS*, minimally conscious state; *VS*, vegetative state; *Abx*, antibiotic; *PLS-DA*, partial least squares discriminant analysis; *VIP*, variable importance in projection; *FDR*, false discovery rate.

Some prolonged DoC patients had been using antibiotics for a long time to prevent infection because of tracheotomy or hypostatic pneumonia (36). To explore the effects of antibiotics on the intestinal microbiome of patients with prolonged DoC, we compared the fecal microbiota taxonomic structure of the EMCS, MCS, and VS groups with that of the corresponding antibiotic treatment groups. The demographic and clinical characteristics of these cohorts are shown in **Supplementary Table S1**. The PLS-DA model showed a distinct separation of the MCS, VS, and their corresponding antibiotic treatment groups (*R* ^2^ = 0.978, *Q* ^2^ = 0.553) (**Figure 4A**). Interestingly, only the MCS group was significantly distinguished from its corresponding antibiotic treatment group (MCS-Abx) in the PCoA model (**Figure 4B**), but could not be observed in the EMCS and VS groups compared with their corresponding antibiotic treatment groups (EMCS-Abx and VS-Abx, respectively) (**Supplementary Figures S2A, B**). Specifically, we compared the taxonomic distributions of the fecal microbiota at the genus level in the MCS and MCS-Abx groups. The relative abundance of *Enterococcus*, *Dialister*, and *Blautia* showed significant differences between the MCS-Abx and MCS groups (**Figure 4C** and **Supplementary Table S2**), whereas no significant differences between EMCS and EMCS-Abx, as well as between VS and VS-Abx (**Supplementary Figures S2C, D**), were shown, suggesting that the intestinal microbiota of MCS patients were more sensitive to antibiotics than those of EMCS and VS patients. Additionally, both the mean community richness (ACE and Chao1) and microbial diversity (Shannon and Simpson diversity indices) were dramatically decreased in the MCS-Abx group compared to the MCS group (**Figure 4D**), but not in the other groups (**Supplementary Figures S2E, F**).

We found that most intestinal microbiota decreased and only a few (i.e., *Enterococcus* and *Halomonas*) were enriched in the MCS-Abx group, as shown in the heatmap (**Figure 4E**). We also performed MetaStat analysis to explore the most significantly different microbiota between these two groups (**Supplementary Table S3**). Of these, the three groups with the greatest differences included *Enterococcus*, which showed a higher relative abundance in the MCS-Abx group, with *Blautia* and *Dialister* showing the opposite (**Figure 4F**). Accordingly, most of the SCFAs in the fecal samples, including acetic, propionic, isobutyric, butyric, and isovaleric acids, dramatically decreased in the MCS-Abx group (**Figure 4G**) and were not significantly different in the other pairwise comparisons (**Supplementary Table S4**).

### Behavioral Scale and EEG Reactivity Between MCS Patients Treated With and Without Antibiotics

**Figure 5:**
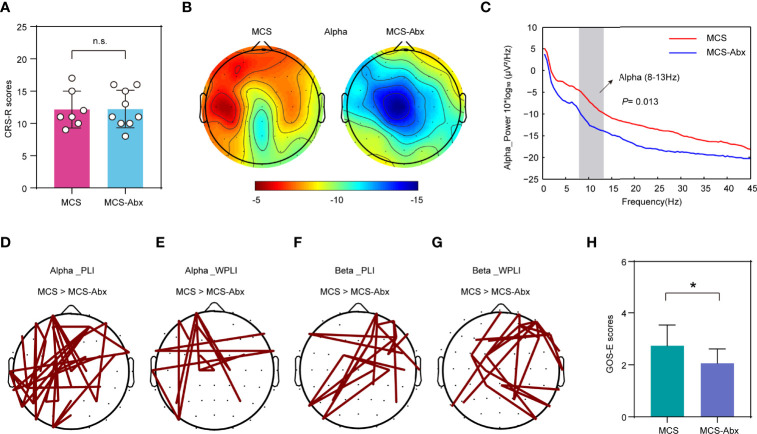
Comparison of the behavioral scale and EEG signals of MCS patients with and without antibiotic treatment. **(A)** Comparison of the CRS-R scores of the patients with EEG recordings in the MCS and MCS-Abx groups. Data represent the mean ± SD. **(B)** Topography of the average degree of the alpha band power for the two groups. *Red* indicates greater alpha band power. **(C)** Spectral power in the MCS and MCS-Abx groups. Averaged alpha power for the differential electrodes (number of sites: 3, 5, 6, 11, 17, 18, 20, 21, 24, 28, 30, 31, 36, 38, 42, 44, and 48). The *vertical gray bar* highlights the portion of the graph corresponding to the alpha frequency ranges, both of which showed a significantly decreased power in the MCS-Abx group (*p* = 0.013). The *red line* denotes the MCS group and the *blue line* indicates the MCS-Abx group. **(D–G)** Connected topoplot of the PLI and WPLI between the MCS and MCS-Abx groups in the alpha and beta bands. NBS was used to identify the interregional connectivity that significantly differed between the groups for the alpha and beta bands. *Red lines* indicate higher connectivity in the MCS group than that in MCS-Abx group. **(D)** PLI in the alpha band (*p* = 0.010). **(E)** WPLI in the alpha band (*p* = 0.039). **(F)** PLI in the beta band (*p* = 0.002). **(G)** WPLI in the beta band (*p* = 0.006). **(H)** Comparison of the GOS-E scores followed for 6 months between the MCS and MCS-Abx groups. Data represent the mean ± SD. *P*-values were determined using Student’s *t*-test or the Kruskal–Wallis test and were corrected using FDR. *p < 0.05; *n.s.*, no significant difference. *PLI*, phase lag index; *WPLI*, weighted phase lag index; *NBS*, network-based statistic; *CRS-R*, Coma Recovery Scale—Revised scores; *GOS-E*, Glasgow Outcome Scale—Extended; *MCS*, minimally conscious state; *Abx*, antibiotic; *FDR*, false discovery rate.

Subsequently, we examined the differences of the brain function and prognosis of MCS patients treated with and without antibiotics. In this study, the CRS-R scores of all patients showed no significant differences between the prolonged DoC groups and their corresponding antibiotic treatment groups (**Supplementary Table S1**). To determine more objective evaluation indicators, we investigated the resting EEG spectral power and functional connectivity from a portion of the patients with intact skulls on the day of fecal collection. As expected, there were no significant differences in the CRS-R scores between the patients in the MCS group and those in the MCS-Abx group with intact skulls and who were selected for EEG recording (**Figure 5A**). Interestingly, patients in the MCS group showed higher power of the alpha band in the left hemisphere compared to those in the MCS-Abx group, especially in the frontal and parietal regions (**Figure 5B**). Furthermore, we screened out the relevant differential electrodes and found that their alpha power values were significantly lower in the MCS-Abx group (**Figure 5C**), suggesting that long-term antibiotic use may weaken the alpha band in the frontal parietal lobe of MCS patients. To better measure the differences between the two groups, we assessed functional connectivity including PLI and WPLI, which are used to evaluate the connectivity between brain regions that share functional properties (37) and are decreased in many neurological diseases (38, 39). Using the NBS approach and a connected topoplot, we observed that the PLI and WPLI were significantly higher in the MCS group than in the MCS-Abx group in the alpha band. The significantly stronger connections were mainly located in the left frontal–occipital site. In addition, these two indicators in the MCS group were also prominently higher in the beta band, and the connection of the right frontal to the left occipital was the most significant (**Figures 5D–G**). To further investigate the effects of long-term antibiotic use on the prognosis of MCS patients, we compared their prognostic scores followed for 6 months and found that the GOS-E scores of the MCS group were higher than those of the MCS-Abx group (**Figure 5H**), suggesting that long-term antibiotic use may cause poor prognosis in these patients.

### Identification of Gut Microbiome-Related Biomarkers to Distinguish EMCS, MCS, and VS Patients

**Figure 6:**
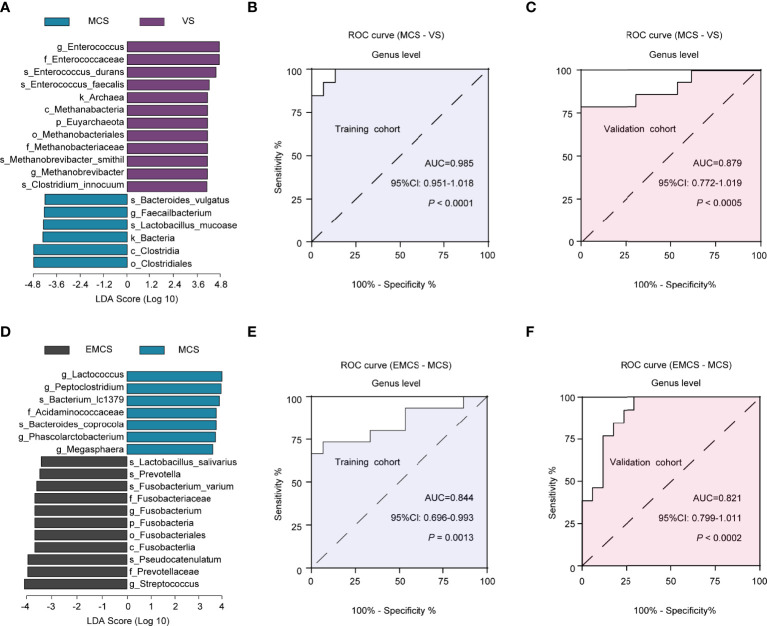
Linear discriminant analysis (LDA) effect size (LEfSe) method used for the altered fecal microbiota and the identification of candidate biomarkers in the discovery and validation cohorts. **(A)** The LDA scores showed significant differences in the microbiota composition between the MCS and VS groups. Only those taxa with an LDA threshold >4 are shown. **(B, C)** ROC curves for *Enterococcus*, *Methanobrevibacter*, and *Faecalibacterium* used in distinguishing between MCS and VS patients in the discovery cohort **(B)** and in the validation cohort **(C)**. **(D)** The LDA scores showed significant differences in the microbiota composition between the EMCS and MCS groups. Only those taxa with an LDA threshold >3.5 are shown. **(E, F)** ROC curves for *Lactococcus* and *Streptococcus* used in distinguishing EMCS from MCS patients in the discovery cohort **(E)** and in the validation cohort **(F)**. *ROC*, receiver operating characteristic; *AUC*, area under the ROC curve; *EMCS*, emerged from minimally conscious state; *MCS*, minimally conscious state; *VS*, vegetative state.

To evaluate the diagnostic efficacy of the intestinal microbiome in patients with different levels of consciousness, LEfSe analysis was conducted to determine and distinguish the composition of the intestinal microbiota between the EMCS and MCS groups and between the MCS and VS groups. The intestinal microbiota of the patients in the MCS group was dominated by the genus *Faecalibacterium*, whereas the microbiome of the patients in the VS group was dominated by the genera *Enterococcus* and *Methanobrevibacter* (**Figure 6A**). We established a diagnostic panel of these three genera to distinguish MCS patients from VS patients, which were all included in the model using the LASSO algorithm. Thereafter, we assessed their diagnostic performance using ROC analysis with an AUC of 0.985 (cutoff = 0.548, *p* < 0.0001, 95%CI = 0.951–1.018) (**Figure 6B**). To validate the candidate biomarkers, they were tested in the validation cohorts (**Supplementary Table S5**), the results of which showed that they achieved high accuracy (AUC = 0.879, cutoff = 0.443, *p* < 0.0005, 95%CI = 0.772–1.019) (**Figure 6C**). When the EMCS group was compared with the MCS group, it was found that the intestinal microbiota of the former was dominated by *Streptococcus* and *Fusobacterium* at the genus level, whereas the microbiome of the latter was dominated by the genera *Lactococcus*, *Peptoclostridium*, *Phascolarctobacterium*, and *Megasphaera* (**Figure 6D**). The combination of *Streptococcus* and *Lactococcus* showed the highest LDA scores distinguishing patients in the EMCS group from those in the MCS group. Finally, the AUCs were 0.844 (cutoff = 0.609, *p* = 0.0018, 95%CI = 0.696–0.993) in the discovery cohort and 0.821 (cutoff = 0.434, *p* = 0.0002, 95%CI = 0.799–1.011) in the validation cohort using the LASSO algorithm and ROC analysis (**Figures 6E, F**). The discrimination validity was also examined using support vector machines. However, the AUCs were 0.654 for MCS to VS patients and 0.733 for EMCS to MCS patients (**Supplementary Figures S3A, B**), which were not better than those of the previous method.

To further investigate whether this panel can be extended to the antibiotic cohort, we performed the LASSO algorithm and ROC analysis in the antibiotic groups. The results revealed AUC values of 0.895 (cutoff = 0.017, *p* = 0.0011, 95%CI = 0.758–1.033) between the MCS-Abx and VS-Abx groups (**Supplementary Figure S3C**) and 0.647 (cutoff = 0.821, *p* = 0.2235, 95%CI = 0.420–0.875) between the EMCS-Abx and MCS-Abx groups (**Supplementary Figure S3D**).

## Discussion

In this study, we identified specific signatures of the fecal microbiota among patients with different levels of consciousness. We found that the altered intestinal microbiota was significantly correlated with the clinical behavioral scores (CRS-R scores) of the patients and the concentrations of SCFAs in their fecal samples. In the comparison of the groups with and without antibiotic treatment, only the MCS-Abx group showed dramatically different fecal microbiota and SCFAs relative to the MCS group, which is consistent with the EEG reactivity of the patients. Finally, we identified potential diagnostic biomarkers for distinguishing patients with different levels of consciousness both in the discovery and validation cohorts, which can also be extended to the antibiotic cohort. All of these findings indicate that alterations of the intestinal microbiota may play an important role in the identification of patients with different levels of consciousness and may influence the recovery of brain function in these patients, especially for MCS patients. To our knowledge, this is the first investigation of the intestinal microbiome in prolonged DoC patients and exploration of the effects of antibiotics on their intestinal microbiota and brain function. This study may provide a new perspective for the evaluation and treatment of patients with prolonged DoC.

Investigation of the intestinal microbiome can help explore its association with patients with different levels consciousness and provide a reference for nutrition-related interventions. All of the patients in our study received enteral nutrition from the hospital, which minimized the bias for gut microbiomes and lipid metabolism caused by different dietary habits. Different from that of TBI patients in the acute stage (40), the overall diversity and community richness of the intestinal microbiota were not altered among patients in the EMCS, MCS, and VS groups, and the same results have been shown in those who had experienced mild or moderate/severe TBI several years ago, but who did not fall into prolonged DoC (41, 42). However, the structure of the intestinal microbiota of the three groups was shown to be dramatically different. A few beneficial microbial communities, such as *Bacteroides*, *Streptococcus*, and *Roseburia*, were enriched in EMCS patients, whereas some pathogenic microbial communities were enriched in VS patients, which indicated that the intestinal microecology might not return to its original state; however, a new dynamic balance was established when severe DoC patients entered into a chronic stage. Currently, only a few probiotics including *Lactobacillus* or *Bifidobacterium* are commonly used for a number of neurological diseases in the clinic (43, 44). More specific probiotics should be developed and available for prolonged DoC patients. Furthermore, the levels of acetic, propionic, and butyric acids, which comprise more than 95% of the total SCFA pool (10, 34) and are produced primarily from dietary fiber, were decreased in VS patients. A large number of studies have shown that SCFAs regulate a growing list of physiological and biochemical functions of the host, including the gut–brain axis (45) and immunological function (46). In our previous metabolomics study, we found that most of the metabolites containing SCFAs were also decreased in the plasma of VS patients (19). Therefore, it is essential to supplement sufficient SCFAs and dietary fiber for these patients. More importantly, most of the SCFAs are produced by the intestinal microbiota and can influence the brain (47). In our results, some of the differential SCFAs in the three groups also comprised the differential microbiota in these patients. For example, *Lactobacillus* can produce acetic acid (48), and *Roseburia* can influence the production of propionic and butyric acids (47). Additionally, some differential microbiota showed a high correlation with SCFAs, as well as with the CRS-R scores of the patients, which suggested that disturbance of the intestinal microbiota and reduction of the corresponding SCFAs may influence the recovery of consciousness after brain injury.

In our study, the patients in the antibiotic cohort had been administered piperacillin or cefoperazone for a long time. Interestingly, only the intestinal microbiome of the MCS patients was significantly altered relative to those who did not receive antibiotic treatment. Broad-spectrum antibiotics may decimate the intestinal microbiome (49). Although some pathogenic microbial communities, such as *Desulfovibrio* and *Macrococcus*, were significantly inhibited, a large number of beneficial bacteria accompanied by the production of SCFAs decreased sharply, which was not conducive to the recovery of the brain function of patients. A previous study demonstrated that antibiotics may influence the brain function through the gut–brain axis (50, 51), but they may also induce impaired consciousness in some patients with metabolic disorders (52, 53). In our study, the power of the alpha band in the antibiotic cohort was decreased in the frontal and parietal regions. The alpha band is considered to have an active role in network coordination and communication (54), and an increase of power and coherence of the frontal and parietal alpha frequency band has been observed when the brain of MCS patients was activated (55). These brain regions are critical areas of the default mode network (DMN), which has been shown to reflect the level of consciousness of DoC patients (56). Therefore, it is possible that the DMN is indirectly disrupted by antibiotics, thereby affecting the recovery of patients’ consciousness. On the other hand, the brain network was damaged and the prognosis was poor in the antibiotic cohort, reflected in the weakened functional connectivity (PLI and WPLI) and GOS-E scores, suggesting that long-term antibiotic use may affect other consciousness-related networks and patient prognosis, even though this performance could not be observed in the CRS-R scale in time. Moreover, the alteration of the brain functional connectivity and the poor outcomes of MCS patients might explain the loss of abundant beneficial microbial communities caused by antibiotics. However, further work is needed to explain the causal relationship and identify the mechanisms underlying these alterations. In summary, when prolonged DoC patients are administered antibiotics for a long period in order to prevent infection, their intestinal microecology should also be protected.

Another important highlight of our study is that we identified biomarkers from the intestinal microbiome to distinguish between VS and MCS patients and between MCS and EMCS patients. Combining *Faecalibacterium*, *Enterococcus*, and *Methanobrevibacter*, we were able to distinguish MCS patients from VS patients with high accuracy in both the discovery and validation cohorts, and the diagnosis panel can be extended to the antibiotic cohort as well. We also used support vector machines to distinguish the three different levels of consciousness, which all showed higher accuracy, although weaker than that of the LASSO algorithm. In clinical practice, the CRS-R scale is commonly used for the diagnosis of VS or MCS patients, but lacks sufficient accuracy (57, 58). Although electrophysiology and imaging can also improve the accuracy (59–61), it is difficult to generalize their use due to the metal implants and incomplete skulls of a lot of patients. Our diagnostic indicators have some advantages, including being noninvasive and convenient to use. We also investigated potential biomarkers to distinguish EMCS patients from MCS and found two indicators with sufficient accuracy. However, this study also has some limitations. Firstly, it described the differences in the intestinal microbiome and brain function of patients with and without antibiotic treatment, but did not reveal the causal effect. We also cannot exclude some variations for the data from systemic inflammation, although there were no differences in the C-reactive protein levels in the different groups. Some variations from the diets may also influence the gut microbiomes of patients, although they were found to be similar. In addition, the sample size is limited, and the conclusion should be replicated in an independent study with a large sample size in the future.

## Conclusion

In summary, we found that patients with different levels of consciousness have different intestinal microbiomes and SCFAs, which may influence brain function, especially in MCS patients. Moreover, *Faecalibacterium*, *Enterococcus*, and *Methanobrevibacter* were considered as potential biomarkers distinguishing MCS from VS patients with high accuracy both in the discovery and validation cohorts.

## Data Availability Statement

The datasets presented in this study can be found in online repositories. The names of the repository/repositories and accession number can be found below: NCBI, PRJNA813677.

## Ethics Statement

The studies involving human participants were reviewed and approved by the Ethics Committee of the First Affiliated Hospital, School of Medicine, Zhejiang University. The patients/participants provided written informed consent to participate in this study.

## Author Contributions

BL and JY conceived the project. JY, ML, and BL designed the experiments. JG, JL and LH screened the patients. JY, YY, XW, and QC collected and repackaged the stool samples. FH extracted the genome DNA. JY and FM completed the microbiome analysis. JY, CX, and GP completed the EEG analysis. JY, ML, and BL wrote the manuscript. All authors discussed, reviewed, and edited the manuscript. All authors contributed to the article and approved the submitted version.

## Funding

This work was supported by grants from the Natural Science Foundation of China (nos. 82071173 and 81901068). This work was also supported by the Key Realm R&D Program of Guangzhou (202007030005).

## Conflict of Interest

The authors declare that the research was conducted in the absence of any commercial or financial relationships that could be construed as a potential conflict of interest.

## Publisher’s Note

All claims expressed in this article are solely those of the authors and do not necessarily represent those of their affiliated organizations, or those of the publisher, the editors and the reviewers. Any product that may be evaluated in this article, or claim that may be made by its manufacturer, is not guaranteed or endorsed by the publisher.

## Supplementary Material

Supplementary Figure 1 Beta diversity and clustering analysis of the fecal microbiota among patients with EMCS, MCS and VS. Related to . **(A)** Beta diversity results of the three groups were assessed by principal coordinate analysis (PCoA); A total of 43 samples and used for PCoA. Two PCs explain 22.1% (18.4% and 13.7%) of variance among the groups (Bray-Curtis distances: EMCS vs. VS: R= 0.1938, P=0.004; EMCS vs. MCS: R= 0.0540, P= 0.064; MCS vs. VS: R= 0.1869, P= 0.003). PC scores are indicated as %; circles indicate individual samples from EMCS, MCS and VS groups. **(B)** Heatmap of the most abundant metabolites among the three groups, as identified by VIP scores in PLS-DA. Each sample represents a single column. Red color indicates the greater abundance of metabolite. PLS-DA, Partial least-squares discriminant analysis; VIP, variable importance in projection; EMCS, Emerged from Minimally Conscious State; MCS, minimally conscious state; VS, vegetative state.

Supplementary Figure 2 Comparison of the fecal microbial diversity and community structures in VS and EMCS patients treated with or without antibiotics. Related to. **(A)** Beta diversity results of the VS and VS-Abx groups were assessed by principal coordinate analysis (PCoA); A total of 33 samples and used for PCoA. Two PCs explain 35.1% (24.1% and 11.0%) of variance between the groups (Bray-Curtis distances: VS vs. VS-Abx: R= 0.0059, *P*=0.453). PC scores are indicated as %; circles indicate individual samples from VS and VS-Abx groups. **(B)** Beta diversity results of the EMCS and EMCS-Abx groups were assessed by PCoA; A total of 24 samples and used for PCoA. Two PCs explain 28.5% (17.2% and 11.3%) of variance between the groups (Bray-Curtis distances: EMCS vs. EMCS-Abx: R= 0.1336, P=0.063). PC scores are indicated as %; circles indicate individual samples from EMCS and EMCS-Abx groups. **(C, E)**: Taxonomic distributions of fecal microbiota of genus level in VS and VS-Abx groups, EMCS and EMCS-Abx groups. **(D, F)** The α-diversity of the fecal microbiome between VS and VS-Abx groups, EMCS and EMCS-Abx groups depict according to ACE, Chao 1, Shannon index and Simpson index. Each box plot represents the median, interquartile range, minimum, and maximum values. n.s., no significant difference; EMCS, Emerged from Minimally Conscious State; VS, vegetative state; Abx, treated with antibiotics.

Supplementary Figure 3 ROC analysis of the candidate biomarkers in the antibiotic cohorts. **(A)** Matrix Diagram and AUC calculated by support vector machine between MCS and VS groups. **(B)** Matrix Diagram and AUC calculated by support vector machine between EMCS and MCS groups. **(C)** ROC curves for *Enterococcus*, *Methanobrevibacter* and *Faecailbacterium* for use in distinguishing MCS patients and VS patients in discovery cohort; **(D)** ROC curves for *Lactococcus* and *Streptococcus* for use in distinguishing MCS patients and VS patients in antibiotics cohort. AUC, area under the receiver-operating characteristic curve. EMCS, Emerged from Minimally Conscious State; MCS, minimally conscious state; VS, vegetative state; Abx, treated with antibiotics.

## Abbreviations

EMCS, emerged from minimally conscious state; MCS, minimally conscious state; VS, vegetative state; GCS, Glasgow Coma Scale; CRS-R, Coma Recovery Scale—Revised scores; GOS-E, Glasgow Outcome Scale—Extended; GPT, glutamic–pyruvic transaminase; GOT, glutamic–oxaloacetic transaminase; LDH, lactate dehydrogenase; TBI, traumatic brain injury; Abx, antibiotic.
