# Supplementary figures and images for: Altered Intestinal Microbiomes and Lipid Metabolism in Patients With Prolonged Disorders of Consciousness
Source: Front Immunol. 2022 Jul 13;13:781148. doi: 10.3389/fimmu.2022.781148 (PMC9326017; doi:10.3389/fimmu.2022.781148)

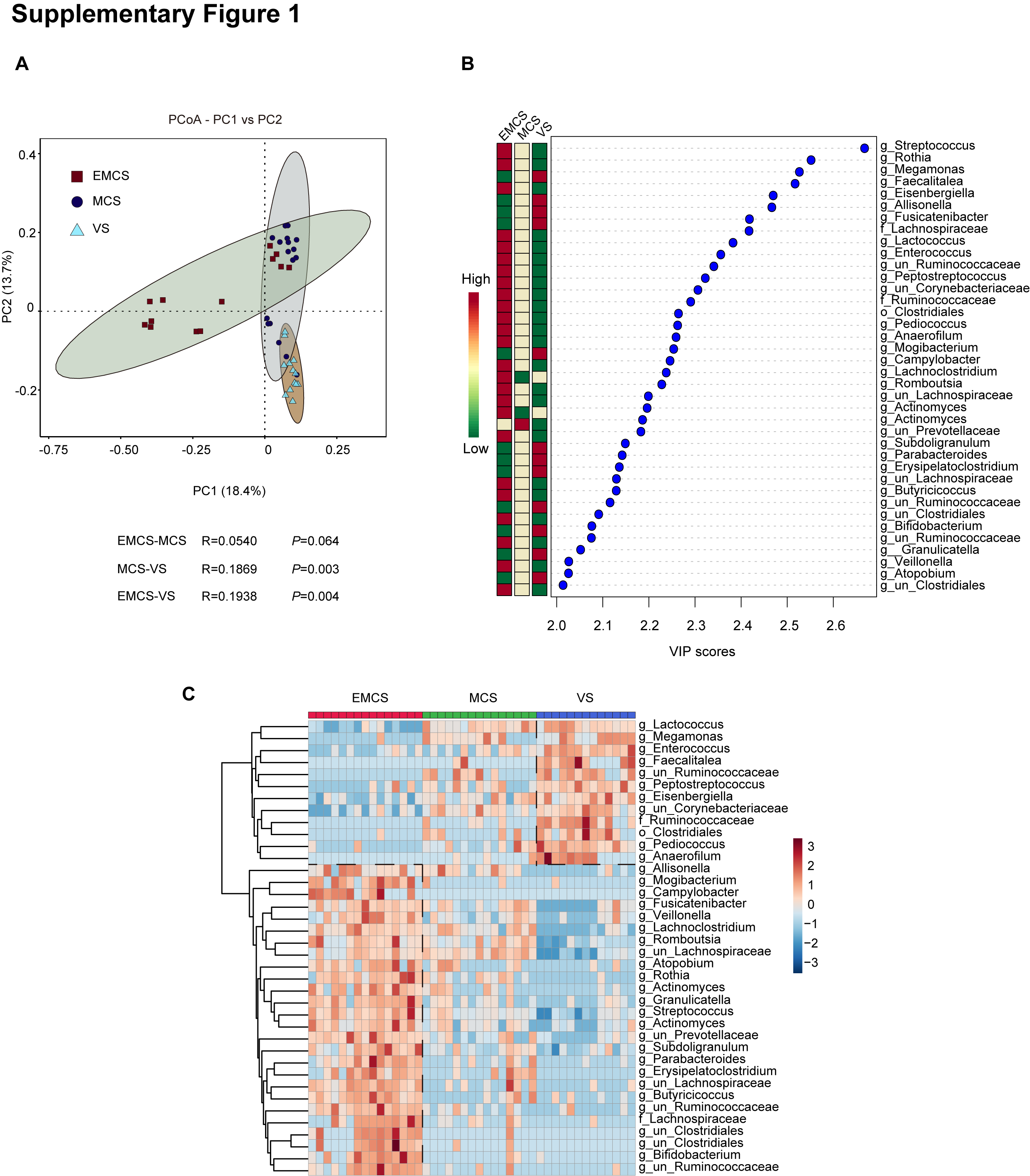

Supplement: Supplementary Figure 1 — Beta diversity and clustering analysis of the fecal microbiota among patients with EMCS, MCS and VS. Related to . (A) Beta diversity results of the three groups were assessed by principal coordinate analysis (PCoA); A total of 43 samples and used for PCoA. Two PCs explain 22.1% (18.4% and 13.7%) of variance among the groups (Bray-Curtis distances: EMCS vs. VS: R= 0.1938, P=0.004; EMCS vs. MCS: R= 0.0540, P= 0.064; MCS vs. VS: R= 0.1869, P= 0.003). PC scores are indicated as %; circles indicate individual samples from EMCS, MCS and VS groups. (B) Heatmap of the most abundant metabolites among the three groups, as identified by VIP scores in PLS-DA. Each sample represents a single column. Red color indicates the greater abundance of metabolite. PLS-DA, Partial least-squares discriminant analysis; VIP, variable importance in projection; EMCS, Emerged from Minimally Conscious State; MCS, minimally conscious state; VS, vegetative state. [file Image_1.tif]

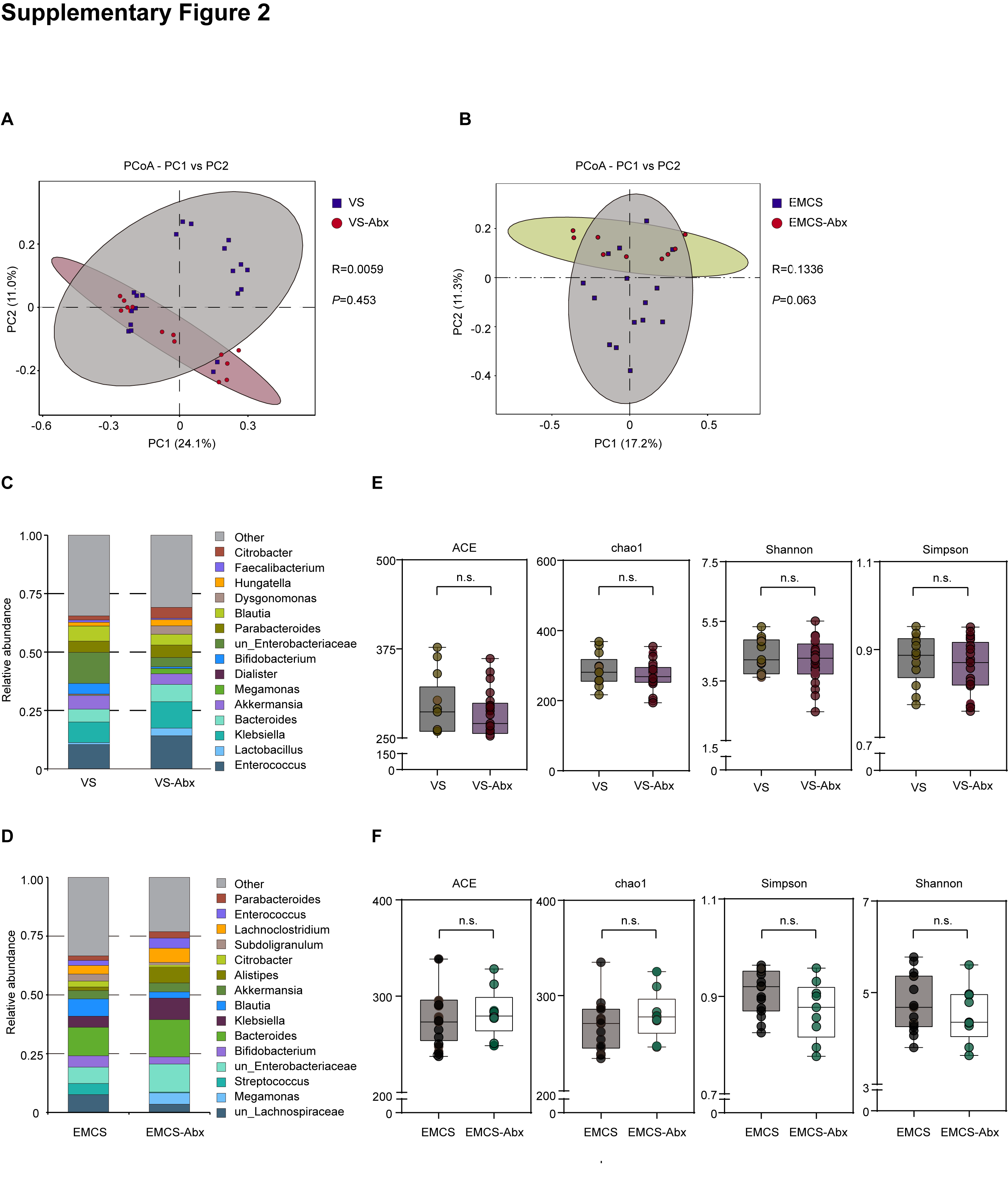

Supplement: Supplementary Figure 2 — Comparison of the fecal microbial diversity and community structures in VS and EMCS patients treated with or without antibiotics. Related to. (A) Beta diversity results of the VS and VS-Abx groups were assessed by principal coordinate analysis (PCoA); A total of 33 samples and used for PCoA. Two PCs explain 35.1% (24.1% and 11.0%) of variance between the groups (Bray-Curtis distances: VS vs. VS-Abx: R= 0.0059, P=0.453). PC scores are indicated as %; circles indicate individual samples from VS and VS-Abx groups. (B) Beta diversity results of the EMCS and EMCS-Abx groups were assessed by PCoA; A total of 24 samples and used for PCoA. Two PCs explain 28.5% (17.2% and 11.3%) of variance between the groups (Bray-Curtis distances: EMCS vs. EMCS-Abx: R= 0.1336, P=0.063). PC scores are indicated as %; circles indicate individual samples from EMCS and EMCS-Abx groups. (C, E): Taxonomic distributions of fecal microbiota of genus level in VS and VS-Abx groups, EMCS and EMCS-Abx groups. (D, F) The α-diversity of the fecal microbiome between VS and VS-Abx groups, EMCS and EMCS-Abx groups depict according to ACE, Chao 1, Shannon index and Simpson index. Each box plot represents the median, interquartile range, minimum, and maximum values. n.s., no significant difference; EMCS, Emerged from Minimally Conscious State; VS, vegetative state; Abx, treated with antibiotics. [file Image_2.tif]

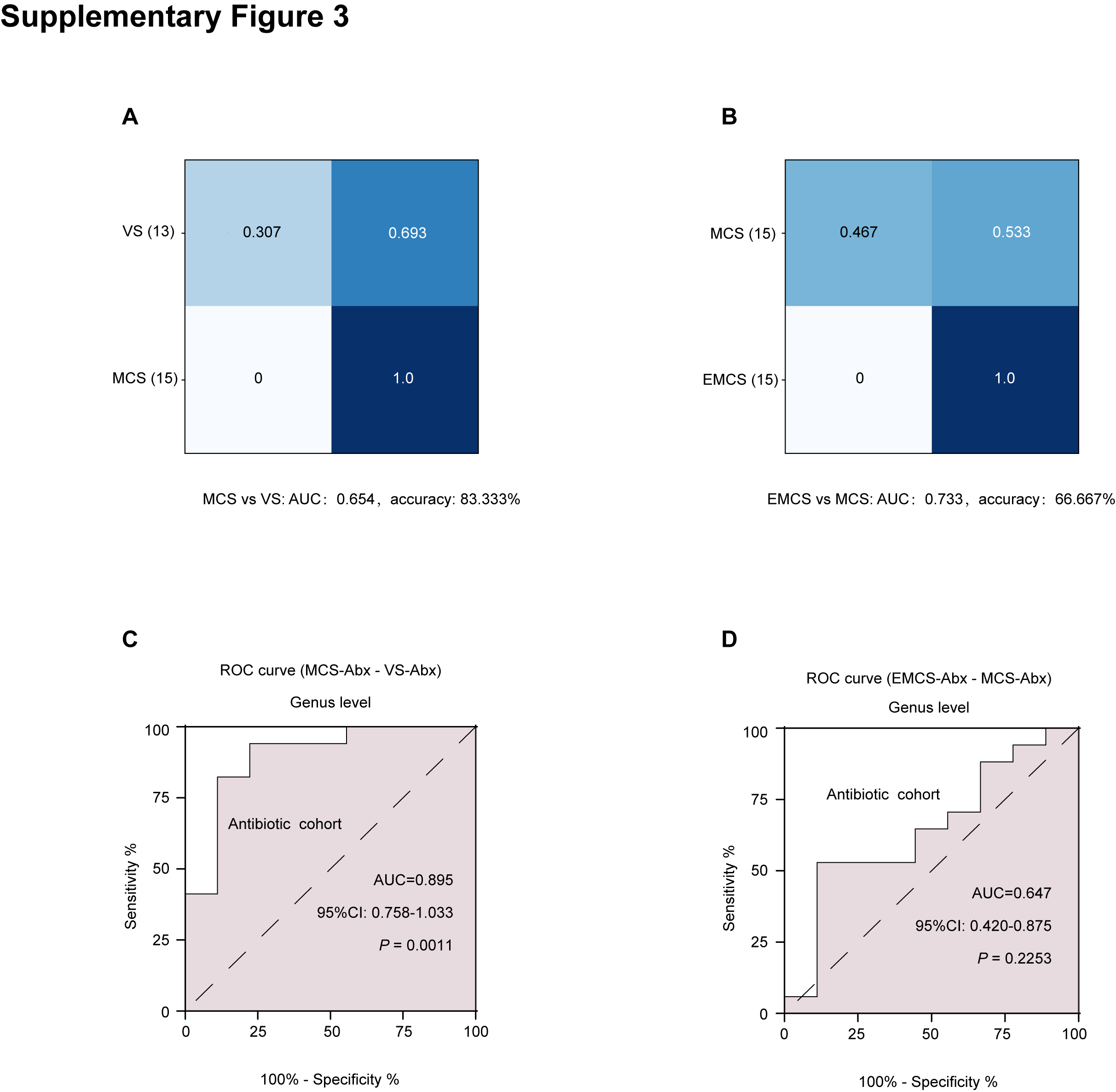

Supplement: Supplementary Figure 3 — ROC analysis of the candidate biomarkers in the antibiotic cohorts. (A) Matrix Diagram and AUC calculated by support vector machine between MCS and VS groups. (B) Matrix Diagram and AUC calculated by support vector machine between EMCS and MCS groups. (C) ROC curves for Enterococcus, Methanobrevibacter and Faecailbacterium for use in distinguishing MCS patients and VS patients in discovery cohort; (D) ROC curves for Lactococcus and Streptococcus for use in distinguishing MCS patients and VS patients in antibiotics cohort. AUC, area under the receiver-operating characteristic curve. EMCS, Emerged from Minimally Conscious State; MCS, minimally conscious state; VS, vegetative state; Abx, treated with antibiotics. [file Image_3.tif]
