# Supplementary material for: Altered Intestinal Microbiomes and Lipid Metabolism in Patients With Prolonged Disorders of Consciousness
Source: Front Immunol. 2022 Jul 13;13:781148. doi: 10.3389/fimmu.2022.781148 (PMC9326017; doi:10.3389/fimmu.2022.781148)
Supplement: Supplementary file 4 [file Table_1.docx]

**Table S1:** Clinical characteristics of patients treated with or without antibiotics.

|  | EMCS vs EMCS-Abx  *P* value | MCS vs MCS-Abx  *P* value | VS vs VS-Abx  *P* value |
| --- | --- | --- | --- |
| Male (n, %) | 0.356 | 0.555 | 0.594 |
| Age (years) | 0.079 | 0.145 | 0.374 |
| GCS | 0.108 | 0.576 | 0.807 |
| CRS-R | 0.815 | 0.823 | 0.129 |
| GOS-E | 0.194 | 0.030* | 0.780 |
| Length of hospital stay | 0.051 | 0.114 | 0.193 |
| Leucocyte (10 ^9^ /L) | 0.453 | 0.589 | 0.484 |
| Neutrophil (10 ^9^ /L) | 0.911 | 0.520 | 0.834 |
| Lymphocyte (10 ^9^ /L) | 0.641 | 0.869 | 0.834 |
| Monocyte (10 ^9^ /L) | 0.079 | 0.261 | 0.567 |
| Hemoglobin (g/L) | 0.217 | 0.001* | 0.218 |
| Platelet (10 ^9^ /L) | 0.685 | 0.073 | 0.527 |
| C-reactive protein (mg/L) | 0.861 | 0.013* | 0.536 |
| GPT (UI/L) | 0.174 | 0.006* | 0.138 |
| GOT (UI/L) | 0.815 | 0.246 | 0.193 |
| LDH (UI/L) | 0.410 | 0.105 | 0.464 |
| Creatinine (μmol/L) | 0.219 | 0.313 | 0.695 |
| Homocysteine (μmol/L) | 0.017* | 0.069 | 0.027* |

Continuous variables are expressed as the mean ± standard deviation (SD); *P* <0.05 was considered statistically significant; GCS: Glasgow Coma Scale; CRS-R: Coma Recovery Scale-Revised scores; GOS-E: Glasgow Outcome Scale – Extended; GPT: glutamic-pyruvic transaminase; GOT: glutamic-pyruvic transaminase; LDH: lactate dehydrogenase; VS: vegetative state; MCS: minimally conscious state; EMCS: Emerged Minimal Conscious state; Abx: antibiotic.

**Table S2:** Comparison of the top15 high abundant fecal microbiota of genus level in MCS and MCS-Abx groups.

|  | MCS  Relative abundance (%) | MCS-Abx  Relative abundance (%) | *P* value (FDR) |
| --- | --- | --- | --- |
| g__Enterococcus | 1.4385 ± 0.2774 | 16.8781 ± 6.2471 | 0.016* |
| g__Lactobacillus | 8.0637 ± 0.0793 | 3.1374 ±0.2370 | 0.567 |
| g__Klebsiella | 7.1832 ± 0.0251 | 16.8796 ± 0.0607 | 0.449 |
| g__Bacteroides | 10.7823 ± 0.0277 | 17.1629 ± 0.0380 | 0.452 |
| g__Akkermansia | 7.4905 ± 0.0017 | 6.3461 ± 0.0338 | 0.866 |
| g__Megamonas | 0.2403 ± 0.0018 | 0.1510 ± 0.0014 | 0.765 |
| g__Dialister | 3.3409 ± 2.8832 | 0.0950 ± 0.0631 | 0.030* |
| g__Bifidobacterium | 2.5832 ± 0.0141 | 6.4541 ± 0.0319 | 0.587 |
| g__un_Enterobacteriaceae | 8.9926 ± 0.0323 | 2.4982 ± 0.0110 | 0.085 |
| g__Parabacteroides | 1.7969 ± 0.0057 | 6.2941 ± 0.0270 | 0.418 |
| g__Blautia | 9.7933 ± 1.9354 | 1.1712 ± 0.3020 | 0.016* |
| g__Dysgonomonas | 0.0132 ± 0.0001 | 0.0334 ± 0.0002 | 0.594 |
| g__Hungatella | 1.2042 ± 0.0055 | 0.3594 ± 0.0011 | 0.194 |
| g__ Faecalibacterium | 2.1555 ± 0.0121 | 1.1820 ± 0.0082 | 0.595 |
| g__ Citrobacter | 1.5290 ± 0.0072 | 0.9410 ± 0.0045 | 0.572 |

Continuous variables are expressed as the mean ± standard error of mean (SEM, %); *P* values are corrected by FDR. FDR: False Discovery Rate. *P* <0.05 was considered statistically significant. MCS: minimally conscious state; Abx: antibiotic.

**Table S3:** MetaStat analysis for different fecal microbiota in genus level between MCS group and MCS-Abx group.

|  | MCS  Relative abundance (%) | MCS-Abx  Relative abundance (%) | *P* value (FDR) |
| --- | --- | --- | --- |
| g__Enterococcus | 1.4385 ± 0.2774 | 16.8781 ± 6.2471 | 0.016 |
| g__Dialister | 3.3409 ± 2.8832 | 0.0950 ± 0.0631 | 0.030 |
| g__Blautia | 9.7933 ± 1.9354 | 1.1712 ± 0.3020 | 0.016 |
| g__Peptoclostridium | 1.3838 ± 0.9977 | 0.0005 ± 0.0005 | 0.016 |
| g__Lactococcus | 1.4926 ± 0.8643 | 0.0437 ± 0.0182 | 0.016 |
| g__Lachnospira | 0.7862 ± 0.4522 | 0.0666 ± 0.0310 | 0.028 |
| g__Desulfovibrio | 0.2438 ± 0.1015 | 0.0324 ± 0.0152 | 0.030 |
| g__Romboutsia | 0.4057 ± 0.0981 | 0.0928 ± 0.0237 | 0.028 |
| g__Anaerostipes | 0.2243 ± 0.0549 | 0.0585 ± 0.0196 | 0.030 |
| g__Cronobacter | 0.0371 ± 0.0192 | 0.0027 ± 0.0010 | 0.016 |
| g__Proteus | 0.0282 ± 0.0144 | 0.0008 ± 0.0004 | 0.016 |
| g__Macrococcus | 0.0120 ± 0.0078 | 0.0001 ± 0.0001 | 0.016 |
| g__Macellibacteroides | 0.0000 ± 0.0000 | 0.0062 ± 0.0062 | 0.016 |
| g__Gemella | 0.0064 ± 0.0017 | 0.0008 ± 0.0003 | 0.016 |
| g__Mailhella | 0.0025 ± 0.0018 | 0.0000 ± 0.0000 | 0.016 |
| g__Caproiciproducens | 0.0013 ± 0.0009 | 0.0000 ± 0.0000 | 0.016 |
| g__unidentified_Bacteria | 0.0017 ± 0.0010 | 0.0002 ± 0.0002 | 0.030 |
| g__Nesterenkonia | 0.0000 ± 0.0000 | 0.0016 ± 0.0008 | 0.016 |
| g__Angelakisella | 0.0011 ± 0.0006 | 0.0000 ± 0.0000 | 0.029 |
| g__Oxalobacter | 0.0011 ± 0.0005 | 0.0000 ± 0.0000 | 0.029 |

Continuous variables are expressed as the mean ± standard error of mean (SEM, %); *P* values are corrected by FDR. FDR: False Discovery Rate. *P* <0.05 was considered statistically significant. MCS: minimally conscious state; Abx: antibiotic.

**Table S4**: Concentration of short chain fatty acid in fecal between EMCS/VS patients with or without antibiotics.

|  | EMCS | EMCS-Abx |  | VS | VS-Abx |  |
| --- | --- | --- | --- | --- | --- | --- |
|  | Concentration (μg/g) | | P value | Concentration (μg/g) | | *P* value |
| Acetic acid | 2475.4±374.7 | 1247.9±377.7 | 0.037* | 1325.9±161.4 | 1760.9±381.4 | 0.845 |
| Propionic acid | 1583.6±225.4 | 984.9±344.3 | 0.144 | 1242.3±171.5 | 1051.9±158.0 | 0.456 |
| Isobutyric acid | 117.6±21.3 | 143.9±16.1 | 0.375 | 190.6±36.6 | 155.1±26.3 | 0.223 |
| Butyric acid | 1135.8±143.2 | 549.4±94.9 | 0.006** | 537.8±90.5 | 505.0±75.9 | 0.793 |
| Isovaleric acid | 124.8±22.7 | 158.8±22.9 | 0.318 | 221.9±39.4 | 169.7±29.1 | 0.307 |
| Valeric acid | 87.8±27.4 | 106.0±16.0 | 0.616 | 94.0±39.5 | 72.6±37.0 | 0.254 |
| Hexanoic acid | 3.8±1.5 | 8.2±3.2 | 0.194 | 5.6±2.4 | 13.8±8.5 | 0.611 |

Continuous variables are expressed as the mean ± standard error of mean (SEM); P <0.05 was considered statistically significant. VS: vegetative state; EMCS: Emerged Minimal Conscious state; Abx: antibiotic.

**Table S5** Clinical characteristics of validation cohorts treated without antibiotics.

|  | EMCS | MCS | VS | *P* value |
| --- | --- | --- | --- | --- |
| Patients(n) | 17 | 13 | 14 | / |
| Male (n, %) | 11(64.7%) | 10(76.9%) | 10(71.4%) | 0.764 |
| Age (years) | 53.0±16.7 | 56.3±14.2 | 57.5±10.5 | 0.906 |
| GCS | 6.7±1.0 | 6.7±0.9 | 6.6±1.2 | 0.788 |
| CRS-R | 22.1±1.5 | 13.1±2.8 | 5.1±1.8 | <0,001* |
| Length of hospital stay | 140.0±59.4 | 145.5±67.9 | 157.8±61.6 | 0.463 |
| Leucocyte (10 ^9^ /L) | 5.9±1.1 | 8.8±7.8 | 6.5±1.4 | 0.553 |
| Neutrophil (10 ^9^ /L) | 3.5±1.3 | 4.2±1.6 | 4.4±1.2 | 0.139 |
| Lymphocyte (10 ^9^ /L) | 1.8±0.4 | 1.4±0.4 | 1.4±0.5 | 0.067 |
| Monocyte (10 ^9^ /L) | 0.5±0.2 | 0.5±0.2 | 0.5±0.1 | 0.749 |
| Hemoglobin (g/L) | 116.8±10.8 | 123.8±18.1 | 113.5±12.7 | 0.075 |
| Platelet (10 ^9^ /L) | 221.1±60.4 | 237.5±88.5 | 233.5±54.5 | 0.626 |
| C-reactive protein (mg/L) | 3.0±3.0 | 8.0±7.5 | 10.4±14.2 | 0.082 |
| GPT (UI/L) | 31.1±18.5 | 38.5±32.0 | 34.9±40.1 | 0.417 |
| GOT (UI/L) | 25.5±15.7 | 28.5±16.5 | 23.1±14.4 | 0.623 |
| LDH (UI/L) | 162.7±33.0 | 186.1±49.0 | 162.4±43.6 | 0.295 |
| Creatinine (μmol/L) | 46.4±12.8 | 46.7±13.9 | 44.6±22.8 | 0.684 |
| Homocysteine (μmol/L) | 8.5±2.6 | 10.9±3.2 | 10.5±2.3 | 0.054 |
| Cause | TBI | TBI | TBI | / |

Continuous variables are expressed as the mean ± standard deviation (SD); *P* <0.05 was considered statistically significant; GCS: Glasgow Coma Scale; CRS-R: Coma Recovery Scale-Revised scores; GPT: glutamic-pyruvic transaminase; GOT: glutamic-pyruvic transaminase; LDH: lactate dehydrogenase; TBI: traumatic brain injury; VS: vegetative state; MCS: minimally conscious state; EMCS: Emerged Minimal Conscious state; Abx: antibiotic.
